# Effects of Different Stroking Styles on Behaviour and Cardiac Parameters in Heifers

**DOI:** 10.3390/ani10030426

**Published:** 2020-03-04

**Authors:** Annika Lange, Sandra Franzmayr, Vera Wisenöcker, Andreas Futschik, Susanne Waiblinger, Stephanie Lürzel

**Affiliations:** 1 Institute of Animal Welfare Science, Department for Farm Animals and Veterinary Public Health, University of Veterinary Medicine, Vienna, Veterinärplatz 1, 1210 Vienna, Austriasusanne.waiblinger@vetmeduni.ac.at (S.W.); stephanie.luerzel@vetmeduni.ac.at (S.L.); 2 Department of Applied Statistics, Johannes Kepler University Linz, Altenberger Str. 69, 4040 Linz, Austria

**Keywords:** human–animal interactions, affective states, positive emotion, expressive behaviour, cattle welfare, stroking, ear positions

## Abstract

**Simple Summary:**

Positive emotions can improve the welfare of animals. Humans can induce positive emotions in cattle via gentle interactions, such as stroking. While previous studies showed that stroking at the lower side of the neck elicited the most positive reactions in cows, cattle groom each other on different body regions and probably react to each other’s signals. We compared the reactions of dairy heifers to two different stroking styles: stroking exclusively on the lower neck or stroking the whole head/neck region and reactively following the signals of the animal. For both styles, we observed longer durations of behaviours indicating positive emotions and relaxation during stroking, suggesting that the animals enjoyed the treatment. The different stroking styles led to differences in the positions of the heifers’ ears: during “reactive” stroking, the animals held their ears longer in low positions, whereas during stroking of the lower neck, the ears spent longer pointing backwards-upwards. However, we did not observe significant differences in other behaviours, indicating that the manner of stroking of the head/neck region seemed to be not very important for the positive perception of stroking. We conclude that both ways of stroking can elicit positive emotions in cattle and increase the animals’ well-being.

**Abstract:**

Gentle animal–human interactions, such as stroking, can promote positive emotions and thus welfare in cattle. While previous studies showed that stroking at the ventral neck elicited the most positive reactions in cows, intra-specific allogrooming in cattle includes different body regions and is probably guided partly by the receiver. Thus, we compared heifers’ (*n* = 28) reactions to stroking with the experimenter either reactively responding to perceived momentary preferences of the heifers or exclusively stroking the ventral neck. Independently of the stroking style, longer durations of neck stretching and contact occurred during stroking, supporting our hypothesis of a positive perception of stroking. We did not confirm the predicted decrease in heart rate and increase in heart rate variability, but instead found a slightly increased mean heart rate during stroking. The different stroking styles elicited differences in the heifers’ ear positions: “reactive” stroking led to longer durations of low ear positions during stroking, while during “ventral neck” stroking, the duration of back up increased. However, no other behaviours differed significantly between different stroking styles, indicating that the exact manner of stroking applied in our treatments seemed to be less important in the promotion of positive affective states in cattle through gentle human–animal interactions.

## 1. Introduction

The promotion of positive emotional states in animals has recently gained more attention in animal welfare science [1,2,3]. The emotional states and welfare of farm animals are strongly influenced by the relationships with the humans they interact with [4,5]. The animal–human relationship is determined by the relative strength of positive and negative emotions of the animal during its interactions with humans [6]. Gentle interactions between humans and animals can induce positive emotions [7,8,9], but are not always effective in doing so, depending on the exact manner of interaction or individual differences [10,11,12] Thus, identifying the characteristics of gentle interactions that enhance their positive perception will contribute to increasing the animals’ wellbeing.

Human–animal interactions can occur through different sensory channels: visual, olfactory, tactile and auditory [6]. Tactile interactions are an important part of the social life of cattle, as evidenced by social licking [13] and its frequent solicitation [14,15]. There is evidence that tactile stimulation is perceived as positive by cattle also if delivered by humans [9,16,17], but the perception is influenced by the body region stroked [9,18].

In several previous studies [19,20] cattle were stroked on the ventral neck, which is the area that led to the most positive reactions compared with stroking of the withers or chest [9]; stroking of the ventral neck improved the animals’ relationship with humans most effectively [18]. Focusing on one defined area allows for higher standardisation of stroking. However, intra-specific allogrooming in cattle includes different body regions [15,21], with animals often moving from one region to another [14], probably at least in part following the receiver’s signals [21,22]. Therefore, reacting in a flexible way to the animal’s behavioural indications of preference, and consequently stroking different body areas, would mimic the social behaviour of cattle more closely and might lead to more positive reactions. While studies in horses have shown that grooming styles reacting to the animals’ signals lead to more positive reactions [23], there is no study investigating reactive stroking treatments in cattle yet.

The effects of human–animal interactions on animal affective states can be assessed using behavioural and physiological indicators [24]. One behaviour that is often observed in cattle during social licking [15,21] and during stroking or brushing by humans is neck stretching [16,17,19], which is therefore interpreted as indicating a positive perception. Recently, facial expressions have been investigated as potential indicators of affective states in animals (reviewed by Descovich et al. [25]). In cattle, ear positions and movements in particular have come into the focus of scientific research on affective states [26,27,28,29]. Social interactions and positive affective states can also affect cardiac parameters. Tactile stimulation in the form of social licking [15] or stroking on the ventral neck [9] has been shown to decrease the heart rate (HR) of cattle. Calculating heart rate variability (HRV) parameters allows for the investigation of changes in sympathetic or vagal activation [30].

We investigated the effects of two different forms of gentle tactile interactions on the behaviour and cardiac parameters of dairy heifers (*n* = 28) habituated to gentle interactions with humans. We compared the heifers’ reactions to exclusively stroking the ventral neck with the reactions to stroking in a reactive way, i.e., with the experimenter including the whole head/neck region and focussing on parts for which the animal indicated a preference.

We hypothesised that both the “ventral neck” and the “reactive” treatment would elicit a positive, low-arousal state in habituated heifers and thus predicted a decrease of HR, an increase of HRV, and an increase of behavioural indicators of low arousal and positive valence. We expected some of these effects to last long enough to be still observed shortly after stroking. Furthermore, we hypothesised that stroking in a reactive way would be perceived as more positive than exclusively stroking the ventral neck. Lastly, we hypothesised that the higher degree of standardisation in the “ventral neck” treatment would lead to a lower variability in the data.

## 2. Materials and Methods

### 2.1. Animals, Housing and Management

The experiment was conducted between May and November 2017 on 28 heifers (27 Austrian Simmental, one Austrian Simmental ×Brown Swiss) kept at the young stock farm (Rehgras, Furth an der Triesting, Austria) of the University of Veterinary Medicine, Vienna. Out of approximately 90 heifers housed there, 32 heifers between 7 and 24 months of age were selected based on their positive animal–human relationship (see Section 2.2). According to their age, they were divided into two stable groups of 16 animals that were housed, fed and treated in the same way; they were kept mainly on pasture and brought into deep litter pens with adjoining outdoor runs only during adverse weather conditions and for testing. There, they were fed hay and a small quantity of concentrate. The animals had ad libitum access to water and mineral blocks.

The experiment was discussed and approved by the institutional ethics committee in accordance with the Good Scientific Practice guidelines and national legislation (project number ETK-02/04/2017).

### 2.2. Selection and Habituation

As we aimed to investigate positive emotions during human–animal interactions, a generally positive perception of close human contact was a prerequisite. The experimenters selected heifers that were actively seeking human contact when approached on pasture and accepted short periods of stroking. Most heifers were already habituated to the experimenters, procedures and equipment from a previous study. The remaining animals were carefully habituated to the camera (SONY HDR-CX730, Weybridge, UK) and HRV equipment (POLAR^®^ Electro Oy, Kempele, Finland), as well as the experimenters (both female, green overalls; stroker: brown hair, 1.63 m; cameraperson: blonde hair, 1.60 m) and the stroking procedure. We used a stepwise habituation approach that developed from letting the heifers explore the experimenters over approaching them while talking in a gentle voice to slowly touching and finally stroking them. We aimed to stop the interaction before the animals showed any sign of avoidance. If needed, concentrate was provided as a food reward until it was possible to equip the free-moving heifers with the HRV girths and stroke the animals for 3 min without any visible signs of fear or walking away. Animals were considered fully habituated when a full 9-min trial (see Section 2.3, stroking of the ventral neck) could be performed without inducing any avoidance reactions. Tests were performed on 28 of the 32 pre-selected heifers.

### 2.3. Experimental Design

We applied a crossover design, i.e., each animal acted as its own control and was thus subjected to both treatments (see Section 2.4.2). To ensure robustness of the data, each animal experienced each treatment three times in an alternating pattern, i.e., in total six trials (trial numbers 1–6). Each trial consisted of three phases of 180 s (3 min) each: (1) pre-stroking (PRE), where the experimenter stood next to the animal so that baseline values could be recorded; (2) stroking (STR), with the experimenter either stroking in a reactive way (“reactive”) by responding to perceived momentary preferences of the heifer or exclusively stroking the ventral neck (“ventral neck”); and (3) post-stroking (POST), where the experimenter was again standing next to the animal so that possible carry-over effects could be observed. Approximately half of the animals started with “ventral neck” stroking and the other half with “reactive” stroking. The experimenters aimed to balance the order of the treatments over each testing day, but due to trial repetitions, complete balancing was not always possible.

### 2.4. Experimental Procedure

#### 2.4.1. General Procedure

All trials were carried out in a deep litter barn of 182 m^2^ (min. 11 m^2^/animal), which was familiar to all animals. Each animal was prepared and equipped for HRV measurement (POLAR^®^, S810i, Polar Elektro Oy, Kempele, Finland), as described previously [19]. All trials were conducted on lying animals during resting phases to minimise the influence of physical activity on cardiac parameters. Before starting a trial, the handler (i.e., stroker) started a POLAR^®^ monitor and placed it in the pocket of the girth. When an animal had been lying for at least 5 min, the camera operator took up position approximately 2 m from the heifer with the camera approximately at the height of the heifer’s eyes, filming the head/neck region from the heifer’s left side, with special focus on the left eye and ear. The stroker assumed a standing position next to the animal’s left shoulder and started the trial. The stroker wore rubber gloves with a rough surface and applied a constant, previously practiced pressure while stroking at a frequency of 40–60 strokes/min [9].

A trial was stopped after its completion of 9 min or aborted earlier at the occurrence of an event likely to influence the animal’s emotional or physiological state (including obvious distractions, the animal standing up or falling asleep (i.e., sleeping position and eyes closed for >10 s) before or during stroking, self-grooming for more than 10 s, or being chased away or showing other social interactions with a herd member). If a trial was stopped, the experimenters waited for at least 1 h before testing the animal again.

#### 2.4.2. Stroking Styles

In the “ventral neck” treatment, the heifer was stroked only on the ventral neck, as in previous studies [9,19]. In the “reactive” treatment, the stroker included the whole head/neck region, always starting behind the left jaw and following a predetermined route until the heifer showed a behaviour indicating a momentary preference, such as presenting a body part, (partly) closing the eyes, stretching the neck or leaning towards the stroker’s hand. The stroker remained at the indicated area of the head/neck region for as long as the heifer showed the indicative behaviour. In both treatments, the animals were stroked continuously for 3 min with one hand.

### 2.5. Behavioural Observations

All trials were video recorded and the behaviour was analysed with the coding software Interact^®^, version 16.1.3.0. (Mangold International GmbH, Arnstorf, Germany), using focal animal sampling and continuous recording [31]. While it was not possible to conceal the treatment, the observers were blinded to the research questions and hypotheses, as well as to the experimental design; the videos were cut to contain one phase each so that the observers were also blinded towards the sequence of the phases. The observers recorded ear and head positions and movements, as well as other behaviour according to an ethogram (Table 1, for photographs of ear positions, see Supplementary Material, Figure S1). Two trained observers conducted the behavioural observations, where one observer analysed the ear positions (intra-observer reliability (IOR): Cohen’s *κ* = 0.78) and the other observer analysed the other behaviours (IOR: Cohen’s *κ* = 0.89–1.00).

### 2.6. Heart Rate Measurements

Data were error-corrected and processed according to Hagen et al. [33] using the Polar Precision Performance Software, version 4.03.050 (Polar Electro Oy, Kempele, Finland), and Kubios, version 2.0 (Biosignal Analysis and Medical Imaging Group, Department of Applied Physics, University of Eastern Finland, Kuopio, Finland). To account for the respiratory rate, frequency bands were set to 0.04–0.2 Hz for the low frequency band and 0.2–0.58 Hz for the high frequency band [30]. The following parameters were statistically analysed: mean heart rate (HR); time domain: standard deviation of the inter-beat intervals (SDNN) and square root of the mean squared differences of successive inter-beat intervals (RMSSD); frequency domain (using a fast Fourier transform): normalised powers of high (HF) and low frequency (LF), LF/HF power ratio (LF/HF).

### 2.7. Statistical Analysis

#### 2.7.1. Behavioural Data

For the statistical analysis of behavioural data, we used the software package R, version 3.5.2 [34]. The durations of behaviours that occurred often enough to be suitable for analysis were transformed to proportions by dividing them by the total time in which they could be observed. To account for the fact that the ear positions are mutually exclusive and their proportions always amount to one, we tried to fit compositional models but the large amount of zeros led to convergence problems. Therefore, we selected the four ear positions that were observed often enough for statistical analysis (median duration in s (min–max): back up, 122 (0–180); back centre, 8 (0–180); centre, 1 (0–170); forward up, 0 (0–148)). They were analysed using generalised linear mixed models (GLMMs) [35] with a beta error structure and logit link function [36,37] using the package “glmmTMB”, version 0.2.3 [38]. To rule out that values of the responses were exactly zero or one, the response variables were transformed according to (y × (n − 1) + 0.5)/n), where y is the original response and n the number of observations [39]. The hanging ear position and the other down positions did not occur often enough to be evaluated statistically on their own (median duration in s (min–max): hanging 0 (0–2)). Thus, we calculated the variable low by summing up the durations of down positions (hanging + back down + centre down + forward down; summed up to low 0 (0–160)). The result was still dominated by zeros, causing difficulties with the beta error distribution; therefore, it was dichotomised (occurrence: yes/no) and analysed using a GLMM with a binomial structure. The sample sizes for models were 516 total measures made for 28 individuals in a total of 172 trials with 3 phases each. For all full models, we included treatment, phase and their interaction as fixed effects, and individual as well as trial ID (trial number nested in individual) as random effects. Trial ID was included as a random effect to account for the fact that each trial consisted of three phases and thus contributed three data points, where it seemed plausible to assume that there was random variation between the trials. We included random slopes within individual for trial number (to account for possible habituation effects with treatment repetition), treatment and phase to allow their effects to vary between individuals [40]. Due to convergence problems with the model for ear flicking, full statistical analysis of this parameter was not possible and the results were inspected graphically.

Since the “ventral neck” stroking style involved a higher degree of standardisation than the “reactive” stroking style, it seemed plausible that the variation in a given observed behaviour would be smaller in the “ventral neck” treatment than in the “reactive” treatment. We explicitly estimated this potential effect by modelling the precision parameter of the response as a function of treatment in each model. Beta distributions can be characterised by a mean and dispersion parameter describing the variation in the distribution around its mean. However, since the variation in the response is actually inversely related to the dispersion parameter, we henceforth label it a precision parameter since a larger precision parameter means a greater concentration of the variable around its mean. Therefore, with a higher degree of standardisation in “ventral neck” stroking, we expected smaller variation in behaviours, and thus, larger estimated precision parameters.

To avoid cryptic multiple testing [41], we compared each full model with a respective null model that lacked the variables of interest (phase and the interaction of phase and treatment) but was otherwise identical. We used a likelihood ratio test (R function “anova”) for these comparisons. The significance of the individual effects was determined by dropping them one at a time and using a likelihood ratio test to compare the resulting models to the full model [40]. Values of *p* ≤ 0.05 are referred to as significant, and 0.05 < *p* ≤ 0.1 as a trend [42]. If the full–null model comparison was significant, non-significant interactions were removed from the models and reduced models were fitted. The significant main effects of treatment are not discussed, as they were not of interest and thus not part of the full–null model comparisons. We determined 95% confidence limits using the function “simulate.glmmTMB” of the “glmmTMB” package.

We assessed the model stability by comparing the estimates of models based on the full dataset with estimates of models fitted to subsets where the levels of the random effects were dropped one at a time [43]. This revealed a fairly good stability of the models, with the exception of the model for low ear positions: in this binomial model, the exclusion of levels of random effects led to more extreme estimates. However, as the direction of these extreme estimates remained the same as in the model for the full dataset, this did not change our interpretation.

Over-dispersion was not an issue in most of the models (range of dispersion parameters: 0.69–1.16; high values indicate high dispersion). In the case of the two models that were overdispersed (contact and forward up), we report standard errors and *p*-values corrected for overdispersion (based on Wald’s z-approximation, therefore no degrees of freedom are indicated and χ^2^s were replaced by z-values) [44]. 

The variables rumination, changes of ear positions and resting head were not analysed using statistical tests, but inspected graphically, as no directed hypotheses could be formulated based on the available literature, however the behaviours might still be affected by emotional state.

For graphical depiction, we used the R packages “ggplot2” [45] and “cowplot” [46]. Data were depicted as Tukey-style boxplots for each treatment and phase, using the mean values of behaviours per animal (averaged across the three trials per treatment). The bold line corresponds to the median; the lower and upper lines of the box to the first and third quartile, respectively; and the whiskers correspond to the lowest and highest values that were still within 1.5 × interquartile range from the margins of the box. Outliers (all values outside of 1.5 × interquartile range) are depicted as circles.

#### 2.7.2. Cardiac Data

Due to technical failure during HRV recording, we obtained a sample size of 27 animals. Cardiac variables were analysed using linear mixed models (LMMs), including treatment; phase and their interaction; age (days); time of day and its quadratic, third and fourth polynomials; HR (unless it was the response variable) and duration of rumination (s) as fixed effects. Trial number nested in animal nested in housing group was considered as a random effect. Heart rate was included as a fixed effect in order to avoid double presentation of the same findings as it is associated with the HRV characteristics [47,48]. By including HR in the models, the results represent the influence of the other independent factors on HRV parameters independently from their influence on HR. The model assumptions were checked via visual inspection of the residuals, and HR, RMSSD, SDNN, HF and LF/HF ratio were log-transformed to meet the assumption of a normal distribution. Likelihood-ratio tests were used to compare models, including the time of day with the models without time and its polynomials; if the difference was not significant, the time of day was excluded from the model. For further model selection, the Akaike information criterion (AIC) was used. Fixed effects (except for the variables of interest treatment, phase and their interaction) were removed from the model if their removal did not result in an increase of the AIC of the resulting model. A false discovery rate control (FDRC) was performed using the Benjamini-Hochberg correction for multiple testing [49] with *n* = 6 and d = 0.05 for significant differences; d = 0.1 was used for trends. The results of the analysis of covariates are not reported as doing so is beyond the scope of this paper.

## 3. Results

### 3.1. Behaviour during Gentle Interactions

**Figure 1:**
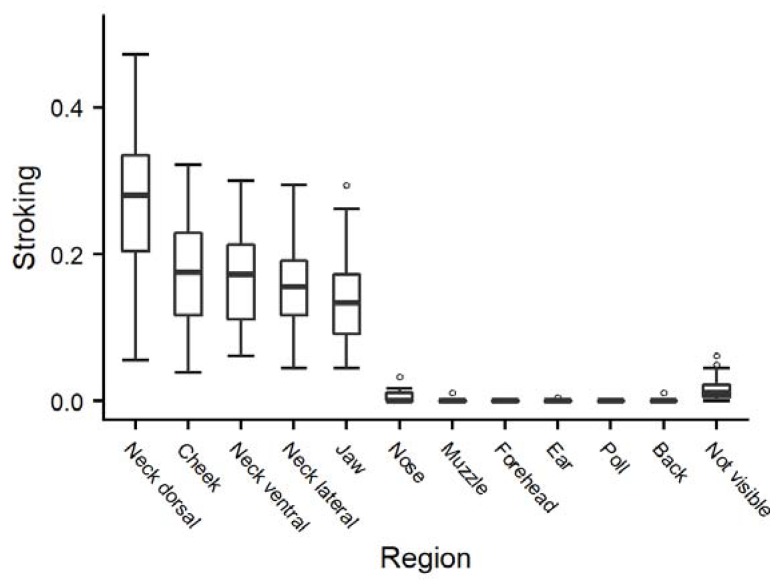
Durations (as a proportion of the total time observed) of stroking for each area of the head/neck region of heifers (*n* = 28) in the “reactive” treatment, averaged across the three trials per animal.

Following the animals’ preferences in the “reactive” stroking style led to varying durations of stroking of the different areas of the head/neck region (Figure 1); the dorsal neck was stroked most for the longest durations, followed by the cheek, ventral neck and jaw, while the muzzle, forehead, ear, poll and back were stroked very rarely.

**Figure 2:**
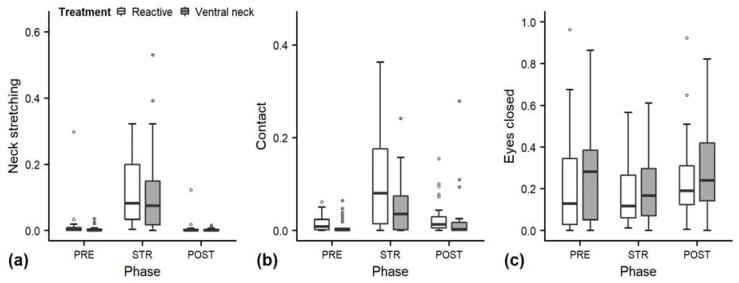
Mean durations (as a proportion of the total time observed) of neck stretching (**a**), contact (**b**) and eyes closed (**c**) of heifers (*n* = 28) during the experimental trials. Means were calculated across the three trials per treatment and are depicted according to the treatment used (white = “reactive”, dark grey = “ventral neck”) and phase (PRE = pre-stroking, STR = stroking, POST = post-stroking). Statistics for GLMMs: significant main effect of phase for neck stretching (**a**) and contact (**b**), *p* < 0.001. Note that the y-axis scale varies to allow for sufficient resolution for rare behaviours.

**Figure 3:**
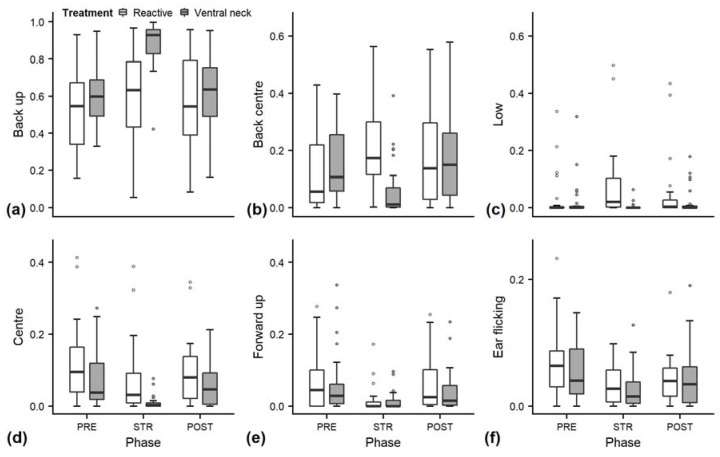
Durations of ear positions of heifers (*n* = 28) (as a proportion of the total time observed) during the experimental trials. Means were calculated across the three trials per treatment and are depicted according to the treatment used (white = “reactive”, dark grey = “ventral neck”) and phase (PRE = pre-stroking, STR = stroking, POST = post-stroking). Statistics for GLMMs: significant effect of treatment × phase for back up (**a**), back centre (**b**) and ear low (**c**), *p* < 0.001; significant main effect of phase: centre (**d**) and forward up (**e**), *p* < 0.05. Ear flicking (**f**) was not evaluated statistically. Note that the y-axis scale varies to allow for sufficient resolution for rare ear positions.

As behaviours indicating positive affective states, we statistically analysed the behaviours neck stretching, contact, eye closed and ear flicking (Figure 2; median duration in seconds (min–max): neck stretching 0 (0–180), contact 0 (0–159), eye closed 12 (0–180); Figure 3f: ear flicking 2 (0–68)).

Full and null models differed significantly for the response variables neck stretching and contact (Figure 2; GLMM: neck stretching: χ^2^ = 28.838, df = 4, *p* < 0.001; contact: χ^2^ = 16.336, df = 4, *p* = 0.003), as well as for all the tested ear positions (Figure 3; back up: χ^2^ = 55.738, df = 4, *p* < 0.001; back centre: χ^2^ = 27.177, df = 4, *p* < 0.001; ear low: χ^2^ = 29.458, df = 4, *p* < 0.001; centre: χ^2^ = 15.010, df = 4, *p* = 0.005; forward up: χ^2^ = 10.294, df = 4, *p* = 0.04). The full–null model comparison revealed no significant difference for eyes closed (χ^2^ = 4.532, df = 4, *p* = 0.3).

Effects of the phase on ear positions depended on the treatment for back up (χ^2^ = 30.100, df = 2, *p* < 0.001), back centre (χ^2^ = 23.835, df = 2, *p* < 0.001), and ear low (χ^2^ = 24.324, df = 2, *p* < 0.001). During “ventral neck” stroking, the durations of back up increased (Figure 3a), durations of back centre (Figure 3b) decreased and durations of ear low did not change substantially, while during “reactive” stroking, no clear changes occurred for back up, and durations of both back centre and ear low increased (Figure 3c).

The interaction of treatment and phase did not have a significant effect on any of the other behaviours. The reduced models for neck stretching and contact revealed a significant main effect of phase (neck stretching: χ^2^ = 27.527, df = 2, *p* < 0.001; contact: z = 2.996, *p* = 0.003), with increases during STR independent of stroking style (Figure 2a,b). Phase also had a significant effect on the ear positions centre and forward up (centre: χ^2^ = 12.350, df = 2, *p* = 0.002; forward up: z = −2.852, *p* = 0.004); during STR, durations of both ear positions decreased independently of stroking style (Figure 3d,e).

The variability was significantly smaller in the “ventral neck” treatment for contact (χ^2^ = 4.851, df = 1, *p* < 0.001) and the ear position centre (χ^2^ = 11.192, df = 1, *p* < 0.001), but higher for neck stretching (χ^2^ = 5.258, df = 1, *p* = 0.022). For statistical details, including model coefficients, standard errors and confidence intervals, see Supplementary Material (Table S1).

**Figure 4:**
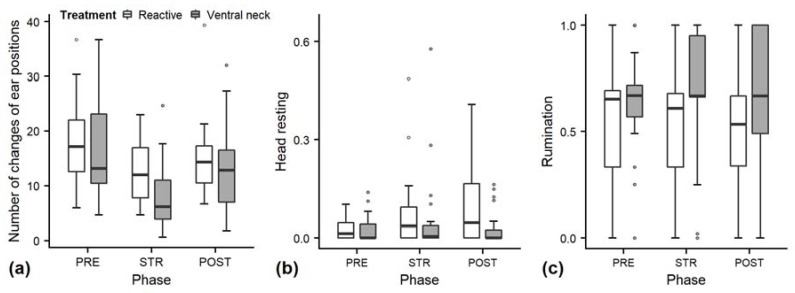
Means of changes of ear position (**a**), head resting (**b**) and rumination (**c**) of heifers (*n* = 28). (**a**): number of changes of ear positions; (**b**,**c**): durations of behaviour as a proportion of the total time observed. Means were calculated across the three trials per treatment and are depicted according to the treatment used (white = “reactive”, dark grey = “ventral neck”) and phase (PRE = pre-stroking, STR = stroking, POST = post-stroking). Note that the y-axis scale varies to allow for sufficient resolution for rare behaviours.

As there were problems with model convergence, ear flicking was only evaluated at the descriptive level, along with head resting, rumination and changes of ear positions (Figure 4). Ear flicking (Figure 3f) and changes of ear positions (Figure 4a) had numerically lower values in STR than PRE and POST. There was no conclusive pattern for the duration of rumination (Figure 4c). The duration of resting head increased numerically over the three phases in the “reactive” treatment but not in the “ventral neck” treatment (Figure 4b). The number of trials stopped because heifers stood up during the stroking phase without any apparent reason was higher in the “ventral neck” treatment (*n* = 7) than the “reactive” treatment (*n* = 4).

### 3.2. Cardiac Data

Phase affected the HR of the animals independently of treatment (Table 2, LMM; χ^2^ = 47.0, df = 2, *p* < 0.05); the HR increased slightly (Table 3, estimated increase of 2.04 bpm in “reactive” compared with 1.60 bpm in “ventral neck”) in STR compared with PRE, but not in POST compared with PRE, according to the contrasts. There was no other significant effect of phase, treatment or the interaction of treatment and phase on any response variable, but a trend towards a main effect of phase on LF/HF with a decrease in STR (Table 2, χ^2^ = 7.0, df = 2, *p* < 0.1).

## 4. Discussion

In line with our hypothesis, different stroking styles (“reactive” vs. “ventral neck”) elicited differences in the heifers’ ear positions. However, no other behaviours differed significantly in reaction to stroking with different stroking styles. Independently of the stroking style, the heifers reacted with longer durations of neck stretching and contact and decreased durations of the ear positions centre and forward up during STR compared with PRE, supporting our hypothesis of a positive perception of stroking. We did not confirm the predicted changes in HR and HRV, but instead found a slightly increased mean HR during stroking, and no changes in HRV parameters.

### 4.1. General Effects of Gentle Tactile Interactions on Behaviour and Cardiac Parameters

We found a significant effect of phase on behaviours indicating a positive affective state during STR for both stroking styles. For instance, the duration of neck stretching increased from PRE to STR. Neck stretching is shown by cattle during intraspecific social grooming [13,21,22] after they actively solicited it, and during stroking by humans [9,19,51] after they voluntarily approached them. It can thus be assumed that the situation is perceived as positive and neck stretching can be interpreted as a sign of enjoyment. The animals also established physical contact with the stroker for longer durations in STR than in PRE. This concurs with other studies where calves leaned against the brush during brushing by a human [17] and heifers approached and proactively offered body parts to a human during positive tactile contact [16]. Following the concept that animals seek out situations of positive valence [2,52,53], seeking proximity to humans indicates that our stroking treatment was perceived as positive.

We expected to induce a low-arousal state during STR. Surprisingly, the mean HR of the animals was significantly higher during STR than PRE; however, the increase was low with less than 2 bpm on average. Although this finding contradicts our hypothesis of a decrease of arousal through stroking, it is in line with the slightly accelerated HR found in animals licked by conspecifics while lying [15]. Since the animals were lying for a minimum of five minutes before we started a trial, we can assume that they were already in a low-arousal state. This is reflected in the low values of baseline HR (raw data, mean ± SD: “reactive” 75 ± 9 bpm, “ventral neck” 77 ± 8 bpm) that were found in PRE and fall below the reported HR of standing cattle that reacted with HR decreases to allogrooming [15,54]. Such low baseline values might have caused a physiological floor effect, where a further decrease of HR is quite impossible, even if stroking is perceived as calming. Additionally, compared to resting in PRE, any physical reaction to the stroking treatment (such as neck stretching, seeking contact to the stroker or presenting body parts) would lead to an increase of HR and might therefore mask the calming effect of stroking. In conclusion, our hypothesis that both stroking styles would elicit a positive, low-arousal state can only be confirmed with regard to valence, but not to arousal. Although there was an effect of phase on HR, there was none on HRV parameters that would have surpassed the effect of the phase on HR. Thus, the stroking of lying heifers did not seem to exert an additional psychophysiological effect on the autonomous nervous system, likely due to the already existing low-arousal, relaxed state and the dominance of vagal regulation during rest [30].

To meaningfully compare our results regarding ear positions with previous findings, differences in the definitions of ear positions need to be considered. Often, specific discrete ear postures are defined [26,32] and their frequency or duration is recorded, which means that ear positions divergent from the predefined postures might not be recorded or analysed. It is not reported which degree of divergence from the definition is allowed for an ear position to still be included in that definition. To cover the continuous spectrum of possible ear positions, we described them according to their position along the vertical and the horizontal axis. This resulted in nine different ear positions: back up, back centre, back down, centre up, centre, centre down, forward up, forward centre and forward down, which were then analysed for their duration, plus ear flicking and ear hanging. This different way of defining ear positions, in our opinion, better reflects the continuous nature of ear positions, but leads to a reduced comparability of our findings with previous studies.

Looking at the proportions of the individual ear positions, we found a decrease of centre and forward up during STR. Erect ears and ears directed forwards have been associated with heightened attention or high-arousal states in dairy cows [28,29]. A decrease of these positions might indicate reduced vigilance or a decrease in arousal during the stroking phase. The graphs show a similar pattern for changes of ear positions and ear flicking, which have numerically lower values during STR. Frequent changes of ear positions were found in reaction to a presumably negative, high-arousal situation in sheep [55] and in dairy cows [27], but also during a positive, presumably low-arousal stroking situation [32]. Ear flicking is a behaviour that is mostly associated with negative affective states [56,57] or reactions to insect attacks [58]. Changes of ear positions and ear flicking should therefore be investigated further as indicators of emotional state.

The effects of the treatment on behaviour and cardiac activity that we saw in STR were not observed in POST, indicating that the positive effects of stroking in lying heifers did not last long enough for carry-over effects to be observed. Some of the observed behaviours (such as neck stretching) are more immediate reactions to positive stimuli and do not allow observation of longer-lasting changes in affective states.

### 4.2. Effects of Stroking Style

Responding to the animals’ signals in the “reactive” stroking style resulted in the longest duration of stroking on the areas of dorsal neck, cheek, ventral neck, lateral neck and jaw (order according to descending duration). This distribution between the neck and head during “reactive” stroking is quite similar to the one found during allogrooming ([21]: neck 65%, head 25% of total duration), which may indicate that the stroker correctly identified the animals’ preferences.

Nevertheless, we found only limited support for our hypothesis that “reactive” stroking would elicit a more positive emotional state than stroking the ventral neck only. The two different tactile stimuli did not lead to significant differences in behaviours or cardiac parameters, except for ear positions. Animals stroked in a “reactive” style showed an increase in low ear positions and in back centre during STR, while animals stroked at the ventral neck showed a significant and strong increase in back up with a concurrent decrease in back centre.

The significant increase of low ear positions during STR in the “reactive” treatment partly confirmed our prediction of lower ear positions during the low-arousal state elicited during STR. However, there was no increase of ear low during “ventral neck” stroking. While we found a similar HR in “reactive” stroking as in “ventral neck” stroking, ear low only increased during “reactive” stroking, possibly indicating that low ear positions are reflecting not only arousal, but must be influenced by other factors as well, such as affective valence or attention. However, low ear positions generally occurred for small proportions of time and far less often than expected. In previous studies, dairy cows showed hanging ears for about 5%–65% of the time [9,32]. Reasons for the short durations of low ear positions, especially ear hanging, in our experiment might be specific to our study population: unlike other studies, which were conducted on adult cattle, we worked with young stock, who might show shorter durations of low ear positions due to a higher reactivity [59]. There are no studies yet investigating the relation of age and low ear positions in cattle.

By far the most common position in our study was the back up position. One factor that possibly influenced the position of the heifers’ ears was the location of the stroker. In our study, the stroker was kneeling beside the animal’s shoulder, possibly causing the heifer to turn her ears backwards and upwards while directing attention to the human. However, the stroker’s position was the same for both stroking styles, but back up increased significantly during the stroking phase only in the “ventral neck” treatment. When interpreting this position, the aforementioned differences in definitions across literature must be taken into account. Backwards ear positions have been found to be associated with both negative and positive affective states. They are part of the facial expressions shown by cattle in pain [60]. However, in a study with a similar design [32], more “back” positions occurred during a low-arousal, positive situation similar to our stroking phase, which might correspond to our result regarding the back up position; however, neither study differentiated the height of the ear in the “back” ear position and it is unclear what position was recorded if the ear was held both backwards and upwards.

One study that defined a position similar to our back up position found higher frequencies during positive states, such as using the brush or feeding compared to queuing to be milked, and suggested it might indicate a higher-arousal positive state than ears back down [26]. The significant increase of back up and the concurrent decrease of back centre that we observed during stroking in the “ventral neck” treatment might thus indicate a higher-arousal state during “ventral neck” stroking than in “reactive” stroking. However, this was not supported by the HR values, which did not differ between the stroking styles. In general, this absence of differences in HR between treatments indicates that the different distributions of ear positions occurring with different stroking styles were probably influenced by factors other than arousal. Therefore, ear positions could be helpful indicators of subtle differences in the valence of affective states of cattle in the future. However, especially regarding the lack of significant differences of HRV or behavioural parameters between the two treatments in our study and the ambiguous findings of previous studies, more research is necessary before making clear interpretations towards the meaning of different ear positions with regard to valence.

One possible alternative explanation for the lack of other differences between the two stroking treatments could be that during “reactive” stroking, the ventral neck was also stroked. An individual might thus have also been stroked mainly at the ventral neck in the “reactive” treatment if it indicated such a preference, which could have led to the absence of obvious differences between the two treatments. However, over all trials of the “reactive” treatment, stroking was performed for the longest durations in the region of the dorsal neck, followed by the cheek, ventral neck, lateral neck, jaw and nose, indicating that there was a meaningful difference between the stroking treatments. Another explanation for the lack of differences lies in a potential masking effect: stroking on the neck might specifically elicit neck stretching as a direct reaction in the case of a positive perception, while stroking another region may induce other behavioural signs of positive perception as well. It is possible that animals perceived the interaction as more pleasant during “reactive” stroking than during “ventral neck” stroking, but because the neck was stroked for shorter durations of the overall treatment time, less neck stretching was induced and similar durations of neck stretching occurred with both stroking styles.

Regarding the behaviours evaluated at the descriptive level, the duration of resting head increased numerically over the three phases in the “reactive” treatment but not in the “ventral neck” treatment, which is comparable with the results of ear low. This behaviour should be recorded and evaluated statistically in future studies.

The conflicting results regarding the variability indicate that the relationship between the degree of standardisation of the treatment and the variability in the observed behaviour is more complex than expected or the standardisation has different effects on different parameters. The higher degree of standardisation in the “ventral neck” treatment did not necessarily lead to a reduction in variability and therefore should not be the sole criterion for the selection of stroking style for gentle human–animal interactions in experimental settings.

## 5. Conclusions

Although we found some differences in ear positions depending on the stroking style, the exact manner of stroking did not have a strong influence on the perception by the animal and thus seems to be less important. Our study supports previous studies indicating that gentle tactile interactions with cattle, provided that the animals have a good relationship with humans, can induce positive emotional states and thus improve their welfare.

## Figures and Tables

**Table 1:** Ethogram adapted from References [9,32].

Behaviour ^(1)^		Definition
Inactive ear posture ^(2)^	Ear hanging	The ear loosely hangs downwards (referring to the ground). There is no visible muscle tension, leading often to a slightly bouncing movement when the position is assumed.
Active ear postures ^(2) (3)^	Back up	The ear is held behind and above the latero-lateral axis.
	Back centre	The ear is held behind at the same height as the latero-lateral axis.
	Back down	The ear is held behind and below the latero-lateral axis.
	Centre up	The ear is held perpendicular to the head and above the latero-lateral axis.
	Centre	The ear is held perpendicular to the head along the latero-lateral axis.
	Centre down	The ear is held perpendicular to the head and below the latero-lateral axis.
	Forward up	The ear is held in front of and above the latero-lateral axis.
	Forward centre	The ear is held in front of and at the same height as the latero-lateral axis.
	Forward down	The ear is held in front of and below the latero-lateral axis.
	Ear flicking	The ear is quickly (within max. 0.5 s) moved back and forth at least once. The behaviour is coded until one of the other ear postures is clearly visible again. The residual movement after the active movement is still part of ear flicking.
Head/neck postures	Held without touching	The head is actively held up and does not touch the stroker.
	Held with touching	The head is actively held up and touches the stroker.
	Rest head without touching	The heifer does not actively carry the head’s weight. The heifer’s head is in contact with the ground, barn equipment, another animal or with the heifer’s leg(s). The heifer’s head is not in contact with the stroker.
	Rest head with touching	The heifer does not actively carry the head’s weight. The heifer’s head is lying on the ground, barn equipment, another animal or the heifer’s leg(s) while being in contact with the stroker, or it is lying on the stroker’s leg.
	Head shaking/tossing	Successive quick movements of the head. The movements can be rotational or up and down.
	Neck stretching	Positioning neck and head actively in an outstretched line, either up, down, or forward.
Eyes ^(4)^	Open	The iris is at least partly visible.
	Closed	The iris is not visible at all for longer than 0.5 s.
	Not visible	Neither eye is visible.
Miscellaneous	Rubbing the stroker	The heifer touches the stroker and moves the touching body part while in contact with the experimenter. The behaviour ends when the contact between the heifer and the person is interrupted for at least 3 s.
	Rubbing	The heifer moves the head/neck region while in contact with the ground or barn equipment. The behaviour ends when the contact between the heifer’s head/neck region and the ground/equipment has ended.
	Nose close	The heifer moves her muzzle towards the stroker within a range of 5 cm. The behaviour ends when the heifer’s nose does not point towards the stroker anymore, leaves the range of 5 cm or if another behaviour of the “miscellaneous” category starts.
	Licking the stroker	The heifer’s tongue touches the stroker at least once. The behaviour ends when the heifer’s tongue does not touch the stroker again within 3 s.
	Ruminating	The heifer’s jaw moves regularly sideways with a frequency of about one movement per second. This movement is recorded as rumination if it occurs in a series of at least five movements (which may start before and end after the observation). Rumination ends when the jaw movement is paused for more than 10 s.
Calculated measures	Contact	The time in which the heifer’s head and neck area was in contact with the stroker. Sum of durations of “rest head with touching”, “held with touching”, “nose close”, “rubbing experimenter” and “licking experimenter”, not including contact established by stroking.
	Resting head	Sum of durations of “rest head with touching” and “rest head without touching”.
	Ear low	The sum of the durations of the ear hanging or held below the latero-lateral axis (“hanging” + “back down” + “centre down” + “forward down”).
	Changes of ear positions	Sum of the frequencies of different ear positions per trial minus 1.

^(1)^ All behaviours were coded as durations, except changes of ear positions (count data). ^(2)^ The left ear was recorded; if it was not visible, the right ear was recorded. ^(3)^ The latero-lateral axis refers to an imaginary line between the bases of the ears. “Behind” means the ear is pointing towards the back of the head, “in front” refers to the rostral end of the head, “above” describes the ear pointing towards the dorsal and “below” towards the ventral part of the head. If the observed ear was moved by the experimenter, the position before the movement was recorded until the next unambiguous ear posture was assumed. ^(4)^ The left eye was recorded; if it was not visible, the right eye was recorded.

**Table 2:** Results of statistical analysis of heart rate (HR) and heart rate variability (HRV) parameters of heifers (*n* = 28). Results that remained significant after false discover rate control (FDRC; *p* < 0.05) appear in bold, trends (*p* < 0.1) in italics; statistics: LMMs. SDNN: standard deviation of the inter-beat intervals, RMSSD: square root of the mean squared differences of successive inter-beat intervals, LF: normalised power of low frequency, HF: normalised power of high frequency.

	Treatment			Phase			Treatment × Phase		
Parameter	χ^2^	df	*p*	χ^2^	df	*p*	χ^2^	df	*p*
HR (bpm)	1.14	1	0.29	**46.99**	**2**	**0.00**	1.67	2	0.43
SDNN (ms)	0.15	1	0.70	1.57	2	0.46	1.88	2	0.39
RMSSD (ms)	0.16	1	0.69	0.78	2	0.68	0.88	2	0.64
LF (ms^2^)	0.18	1	0.68	4.64	2	0.10	0.67	2	0.72
HF (ms^2^)	0.08	1	0.79	5.58	2	0.06	0.54	2	0.76
LF/HF	0.13	1	0.71	*6.99*	*2*	*0.03*	0.95	2	0.62

**Table 3:** Estimated marginal means, standard errors (SE) and lower and upper confidence limits (CL_L_, CL_U_) of HR and HRV parameters of heifers (*n* = 27) for each treatment and phase. PRE = pre-stroking, STR = stroking, POST = post-stroking.

		PRE				STR				POST			
Parameter	Treatment	Mean	SE	CL_L_	CL_U_	Mean	SE	CL_L_	CL_U_	Mean	SE	CL_L_	CL_U_
**HR** **(bpm) ^(1)^**	Reactive	81.1	2.3	56.8	115.8	83.3	2.3	58.3	119.0	81.6	2.3	57.1	116.5
	Ventral neck	83.0	2.3	58.3	118.1	84.7	2.3	59.5	120.5	83.0	2.3	58.3	118.0
**SDNN** **(ms) ^(1)^**	Reactive	27.3	2.0	10.8	68.6	25.6	1.9	10.2	64.3	26.6	1.9	10.6	66.9
	Ventral neck	26.6	1.9	10.9	64.9	25.5	1.8	10.4	62.3	24.2	1.7	9.9	59.0
**RMSSD** **(ms) ^(1)^**	Reactive	14.7	1.3	4.6	46.3	14.1	1.3	4.5	44.3	14.2	1.3	4.5	44.9
	Ventral neck	14.2	1.3	4.7	43.6	13.1	1.2	4.3	40.1	14.0	1.2	4.6	42.8
**HF** **(ms^2^) ^(1)^**	Reactive	12.2	1.4	2.7	54.6	15.2	1.8	3.4	67.5	12.2	1.4	2.7	54.5
	Ventral neck	11.7	1.3	2.8	48.6	14.0	1.6	3.4	58.3	12.6	1.4	3.0	52.1
**LF** **(ms^2^)**	Reactive	71.8	2.5	40.3	103.4	68.9	2.5	37.5	100.3	74.5	2.5	43.1	106.0
	Ventral neck	73.0	2.3	43.2	102.9	68.9	2.4	39.0	98.8	72.8	2.3	43.0	102.7
**LF/HF ^(1)^**	Reactive	5.6	0.8	0.9	36.2	4.3	0.6	0.7	27.3	6.0	0.9	0.9	37.9
	Ventral neck	6.0	0.8	1.0	35.2	4.8	0.7	0.8	27.9	5.6	0.8	1.0	32.6

^(1)^ Back-transformed from log-scale using the R package “emmeans” [50].

## Supplementary Materials

The following are available online at https://www.mdpi.com/2076-2615/10/3/426/s1. Figure S1: Example photographs of ear positions, Figure S2: Example photographs of ear positions with lines indicating (a) the vertical axis (yellow, through the poll and the caudo-ventral edge of the mandible angle) and (b) the horizontal axis (red, between the bases of the ears), Table S1: Full and reduced models for the different behaviours of the heifers (*n* = 28): comparison between the different stroking styles over the three phases.
