# Supplementary material for: Effects of Different Stroking Styles on Behaviour and Cardiac Parameters in Heifers
Source: Animals (Basel). 2020 Mar 4;10(3):426. doi: 10.3390/ani10030426 (PMC7143138; doi:10.3390/ani10030426)
Supplement: Supplementary file 1 [file animals-10-00426-s001.pdf]

animals

# Supplementary Materials: Effects of Different Stroking Styles on Behaviour and Cardiac Parameters in Heifers

**Annika Lange [1,*], Sandra Franzmayr [1], Vera Wisenöcker [1], Andreas Futschik [2], Susanne Waiblinger [1] and Stephanie Lürzel [1]**

[1] Institute of Animal Welfare Science, Department for Farm Animals and Veterinary Public Health, University of Veterinary Medicine, Vienna, Veterinärplatz 1, 1210 Vienna, Austria; s.franzmayr@liwest.at (S.F.); vera.wisenoecker@a1.net (V.W.); susanne.waiblinger@vetmeduni.ac.at (S.W.); stephanie.luerzel@vetmeduni.ac.at (S.L.)

[2] Department of Applied Statistics, Johannes Kepler University Linz, Altenberger Str. 69, 4040 Linz, Austria; andreas.futschik@jku.at

* Correspondence: annika.lange@vetmeduni.ac.at

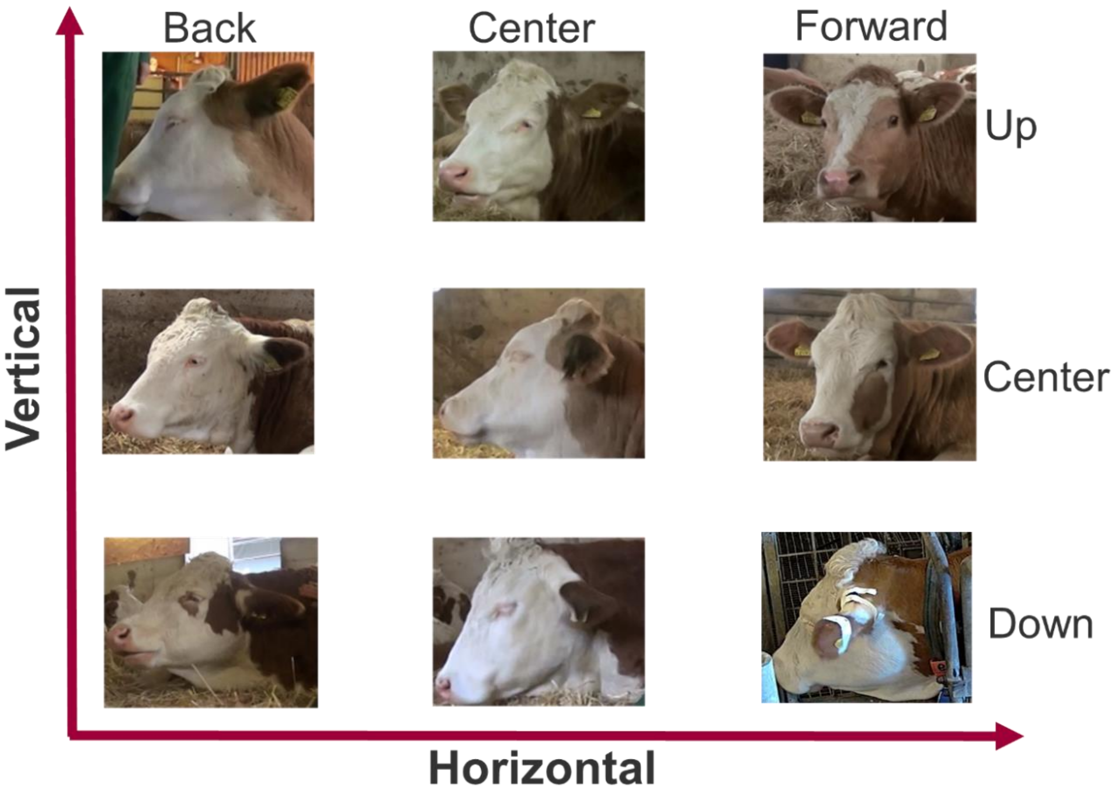


**Figure S1.** Example photographs of ear positions. The ear postures are described relative to the vertical axis, an imaginary line through the poll and the caudo-ventral edge of the mandible angle, and the horizontal axis, an imaginary line between the bases of the ears. "Back" means the ear is pointing towards the back of the head, "forward" refers to the rostral end of the head, "up" describes the ear pointing towards the dorsal and "down" towards the ventral part of the head. The example photograph for "forward down" was taken in another experiment because the position did not occur in our study.

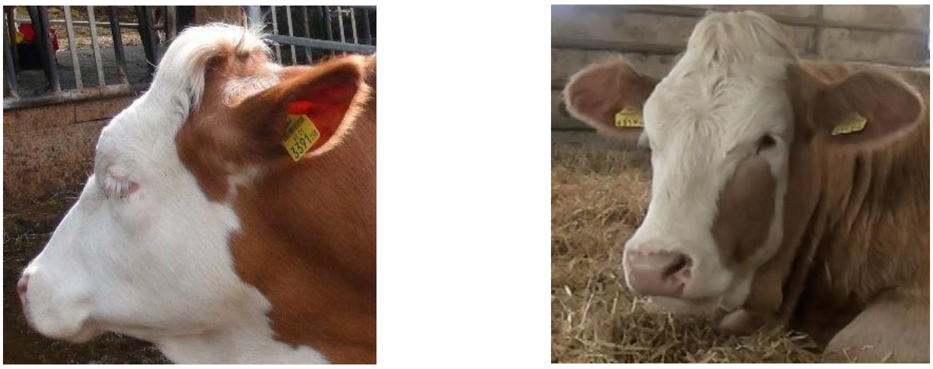

(**a**) (**b**)

**Figure S2.** Example photographs of ear positions with lines indicating (**a**) the vertical axis (yellow, through the poll and the caudo-ventral edge of the mandible angle) and (**b**) the horizontal axis (red, between the bases of the ears).

**Table S1.** Full and reduced models for the different behaviours of the heifers (*n* = 28): comparison between the different stroking styles over the three phases. Statistically significant results appear in bold. CL: confidence limits. Statistics: GLMMs.

| **Full model *neck stretching*** | | | | | | | | |
|---|---|---|---|---|---|---|---|---|
| **Part** | **Effects** | **Coefficients** | **SE** | **$CL_{lower}$** | **$CL_{upper}$** | **$\chi^2$** | **df** | ***p*** |
| Mean | (Intercept) | -3.270 | 0.152 | -3.599 | -3.012 | | | - (1) |
| | Treatment(2) | 0.280 | 0.206 | -0.148 | 0.687 | | | - |
| | Phase (STR)(3) | 0.949 | 0.178 | 0.626 | 1.297 | | | - |
| | Phase (POST)(3) | -0.043 | 0.153 | -0.344 | 0.26 | | | - |
| | Test(2) | 0.005 | 0.043 | -0.082 | 0.095 | 0.013 | 1 | 0.910 |
| | Treatment:Phase(4) | | | | | 1.311 | 2 | 0.519 |
| | Treatment(2) × Phase (STR)(3) | -0.214 | 0.220 | -0.654 | 0.244 | | | - |
| | Treatment(2) × Phase (POST)(3) | 0.011 | 0.218 | -0.401 | 0.469 | | | - |
| Precision | (Intercept) | 1.852 | 0.127 | 1.646 | 2.175 | | | - |
| | **Treatment(2)** | **-0.394** | **0.171** | -0.756 | -0.04 | **5.258** | **1** | **0.022** |
| **Reduced model *neck stretching* without insignificant treatment×phase interaction** | | | | | | | | |
| **Part** | **Effects** | **Coefficients** | **SE** | **$CL_{lower}$** | **$CL_{upper}$** | **$\chi^2$** | **df** | ***p*** |
| Mean | (Intercept) | -3.223 | 0.135 | -3.377 | -2.934 | | | - |
| | Treatment(2) | 0.178 | 0.148 | -0.108 | 0.464 | 1.446 | 1 | 0.229 |
| | **Phase (STR)(3)** | 0.848 | 0.147 | 0.569 | 1.127 | **27.527** | **2** | **< 0.001** |
| | Phase (POST)(3) | -0.037 | 0.109 | -0.250 | 0.177 | | | - |
| | Test(2) | 0.008 | 0.043 | -0.077 | 0.099 | 0.031 | 1 | 0.861 |
| Precision | (Intercept) | 1.831 | 0.125 | 1.634 | 2.119 | | | - |
| | **Treatment(2)** | **-0.349** | **0.167** | -0.663 | -0.014 | **4.345** | **1** | **0.037** |
| **Full model *contact*(5)** | | | | | | | | |
| **Part** | **Effects** | **Coefficients** | **SE** | **$CL_{lower}$** | **$CL_{upper}$** | **$\chi^2$ or z** | **df** | ***p*** |
| Mean | (Intercept) | -2.937 | 0.186 | -3.267 | -2.695 | | | - |
| | Treatment(2) | -0.730 | 0.254 | -1.100 | -0.341 | -2.873 | | 0.004 |
| | Phase (STR)(3) | 0.383 | 0.197 | 0.100 | 0.712 | 1.947 | | 0.052 |

| | | | | | | | | |
|---|---|---|---|---|---|---|---|---|
| | Phase (POST)(3) | 0.084 | 0.197 | -0.210 | 0.390 | 0.429 | | 0.668 |
| | Test(2) | -0.061 | 0.057 | -0.145 | 0.024 | -1.062 | | 0.288 |
| | Treatment(2) × Phase (STR)(3) | 0.054 | 0.272 | -0.382 | 0.479 | 0.197 | | 0.844 |
| | Treatment(2) × Phase (POST)(3) | -0.046 | 0.273 | -0.460 | 0.359 | -0.169 | | 0.865 |
| Precision | (Intercept) | 1.591 | 0.120 | 1.418 | 1.903 | | | - |
| | **Treatment(2)** | **0.833** | **0.172** | 0.504 | 1.198 | **4.851** | **1** | **< 0.001** |
| **Reduced model *contact* without insignificant treatment×phase interaction(5)** | | | | | | | | |
| **Part** | **Effects** | **Coefficients** | **SE** | **$CL_{lower}$** | **$CL_{upper}$** | **$\chi^2$ or z** | **df** | ***p*** |
| Mean | (Intercept) | -2.939 | 0.164 | -3.549 | -3.129 | | | - |
| | Treatment(2) | -0.723 | 0.190 | -1.004 | -0.418 | -3.805 | | < 0.001 |
| | **Phase (STR)(3)** | 0.410 | 0.137 | 0.204 | 0.637 | 2.996 | | **0.003** |
| | Phase (POST)(3) | 0.060 | 0.136 | -0.145 | 0.285 | 0.441 | | 0.66 |
| | Test(2) | -0.060 | 0.057 | -0.147 | 0.028 | -1.055 | | 0.292 |
| Precision | (Intercept) | 1.593 | 0.120 | 1.401 | 1.895 | | | - |
| | **Treatment(2)** | **0.826** | **0.171** | 0.483 | 1.158 | **4.851** | **1** | **< 0.001** |
| **Full model *eyes closed*** | | | | | | | | |
| **Part** | **Effects** | **Coefficients** | **SE** | **$CL_{lower}$** | **$CL_{upper}$** | **$\chi^2$** | **df** | ***p*** |
| Mean | (Intercept) | -1.162 | 0.167 | -1.506 | -0.832 | | | - |
| | Treatment(2) | 0.296 | 0.205 | -0.124 | 0.713 | | | - |
| | Phase (STR)(3) | 0.073 | 0.182 | -0.279 | 0.434 | | | - |
| | Phase (POST)(3) | 0.087 | 0.176 | -0.247 | 0.443 | | | - |
| | Test(2) | 0.102 | 0.084 | -0.064 | 0.279 | 1.446 | 1 | 0.229 |
| | Treatment:Phase(4) | | | | | 2.612 | 2 | 0.270 |
| | Treatment(2) × Phase (STR)(3) | -0.388 | 0.257 | -0.931 | 0.122 | | | - |
| | Treatment(2) × Phase (POST)(3) | -0.062 | 0.251 | -0.576 | 0.461 | | | - |
| Precision | (Intercept) | 0.239 | 0.112 | 0.071 | 0.446 | | | - |
| | **Treatment(2)** | -0.137 | 0.128 | -0.381 | 0.120 | 1.146 | 1 | 0.284 |
| **Full model *back up*** | | | | | | | | |
| **Part** | **Effects** | **Coefficients** | **SE** | **$CL_{lower}$** | **$CL_{upper}$** | **$\chi^2$** | **df** | ***p*** |
| Mean | (Intercept) | 0.191 | 0.178 | -0.196 | 0.514 | | | - |
| | Treatment(2) | 0.181 | 0.190 | -0.191 | 0.559 | | | - |
| | Phase (STR)(3) | 0.241 | 0.161 | -0.086 | 0.549 | | | - |
| | Phase (POST)(3) | 0.070 | 0.151 | -0.220 | 0.363 | | | - |
| | Test(2) | -0.010 | 0.086 | -0.187 | 0.154 | 0.014 | 1 | 0.905 |
| | Treatment:Phase(4) | | | | | **30.100** | **2** | **<0.001** |
| | Treatment(2) × Phase (STR)(3) | 1.167 | 0.233 | 0.693 | 1.578 | | | - |
| | Treatment(2) × Phase (POST)(3) | 0.079 | 0.219 | -0.356 | 0.527 | | | - |
| Precision | (Intercept) | 1.125 | 0.115 | 0.916 | 1.314 | | | - |
| | **Treatment(2)** | -0.149 | 0.139 | -0.395 | 0.112 | 1.166 | 1 | 0.280 |
| **Full model *back centre*** | | | | | | | | |
| **Part** | **Effects** | **Coefficients** | **SE** | **$CL_{lower}$** | **$CL_{upper}$** | **$\chi^2$** | **df** | ***p*** |

| | | | | | | | | |
|---|---|---|---|---|---|---|---|---|
| Mean | (Intercept) | -1.948 | 0.163 | -2.329 | -1.650 | | | - |
| | Treatment(2) | 0.257 | 0.183 | -0.100 | 0.653 | | | - |
| | Phase (STR)(3) | 0.586 | 0.160 | 0.282 | 0.905 | | | - |
| | Phase (POST)(3) | 0.290 | 0.159 | -0.013 | 0.632 | | | - |
| | Test(2) | -0.019 | 0.047 | -0.111 | 0.081 | NA(6) | 1 | NA(6) |
| | Treatment:Phase(4) | | | | | **23.825** | **2** | **<0.001** |
| | Treatment(2) × Phase (STR)(3) | -1.057 | 0.230 | -1.527 | -0.615 | | | - |
| | Treatment(2) × Phase (POST)(3) | -0.189 | 0.228 | -0.645 | 0.272 | | | - |
| Precision | (Intercept) | 1.022 | 0.097 | 0.884 | 1.278 | | | - |
| | **Treatment(2)** | -0.167 | 0.228 | -0.445 | 0.110 | 1.538 | 1 | 0.215 |
| **Full model *centre*** | | | | | | | | |
| **Part** | **Effects** | **Coefficients** | **SE** | **$CL_{lower}$** | **$CL_{upper}$** | **$\chi^2$** | **df** | ***p*** |
| Mean | (Intercept) | -2.101 | 0.138 | -2.398 | -1.860 | | | - |
| | Treatment(2) | -0.566 | 0.183 | -0.929 | -0.219 | | | - |
| | Phase (STR)(3) | -0.203 | 0.157 | -0.510 | 0.108 | | | - |
| | Phase (POST)(3) | -0.099 | 0.156 | -0.414 | 0.219 | | | - |
| | Test(2) | -0.045 | 0.047 | -0.133 | 0.037 | 0.939 | 1 | 0.332 |
| | Treatment:Phase(4) | | | | | 2.660 | 2 | 0.264 |
| | Treatment(2) × Phase (STR)(3) | -0.349 | 0.219 | -0.831 | 0.046 | | | - |
| | Treatment(2) × Phase (POST)(3) | -0.103 | 0.217 | -0.520 | 0.317 | | | - |
| Precision | (Intercept) | 1.254 | 0.108 | 1.087 | 1.515 | | | - |
| | **Treatment(2)** | **0.555** | **3.512** | 0.223 | 0.886 | **11.92** | **1** | **< 0.001** |
| **Reduced model *centre* without insignificant treatment×phase interaction** | | | | | | | | |
| **Part** | **Effects** | **Coefficients** | **SE** | **$CL_{lower}$** | **$CL_{upper}$** | **$\chi^2$** | **df** | ***p*** |
| Mean | (Intercept) | -2.028 | 0.122 | -2.296 | -1.818 | | | - |
| | Treatment(2) | -0.701 | 0.141 | -0.974 | -0.412 | 23.654 | 1 | **< 0.001** |
| | **Phase** | | | | | **12.350** | **2** | **0.002** |
| | Phase (STR)(3) | -0.383 | 0.110 | -0.610 | -0.160 | | | - |
| | Phase (POST)(3) | -0.149 | 0.109 | -0.367 | 0.056 | | | - |
| | Test(2) | -0.048 | 0.047 | -0.143 | 0.045 | 1.066 | 1 | 0.301 |
| Precision | (Intercept) | 1.253 | 0.108 | 1.087 | 1.518 | | | - |
| | **Treatment(2)** | **0.536** | **0.158** | 0.225 | 0.826 | **11.192** | **1** | **< 0.001** |
| **Full model *forward up*(5)** | | | | | | | | |
| **Part** | **Effects** | **Coefficients** | **SE** | **$CL_{lower}$** | **$CL_{upper}$** | **$\chi^2$ or z** | **df** | ***p*** |
| Mean | (Intercept) | -2.809 | 0.143 | -3.132 | -2.567 | | | - |
| | Treatment(2) | -0.091 | 0.197 | -0.487 | 0.309 | -0.461 | | 0.645 |
| | Phase (STR)(3) | -0.283 | 0.154 | -0.585 | 0.054 | -1.835 | | 0.066 |
| | Phase (POST)(3) | 0.046 | 0.153 | -0.255 | 0.361 | 0.301 | | 0.763 |
| | Test(2) | 0.064 | 0.046 | -0.021 | 0.154 | 1.392 | | 0.164 |
| | Treatment(2) × Phase (STR)(3) | -0.057 | 0.216 | -0.517 | 0.372 | -0.264 | | 0.792 |
| | Treatment(2) × Phase (POST)(3) | -0.207 | 0.215 | -0.618 | 0.201 | -0.962 | | 0.336 |

| Part | Effects | Coefficients | SE | $CL_{lower}$ | $CL_{upper}$ | $\chi^2$ or z | df | *p* |
|---|---|---|---|---|---|---|---|---|
| Precision | (Intercept) | 1.620 | 0.125 | 1.441 | 1.938 | | | - |
| | **Treatment[(2)]** | 0.222 | 0.174 | -0.144 | 0.580 | 1.619 | | 0.203 |
| **Reduced model *forward up* without insignificant treatment×phase interaction[(5)]** | | | | | | | | |
| **Part** | **Effects** | **Coefficients** | **SE** | **$CL_{lower}$** | **$CL_{upper}$** | **$\chi^2$ or z** | **df** | ***p*** |
| Mean | (Intercept) | -2.763 | 0.128 | -3.028 | -2.548 | | | - |
| | Treatment[(2)] | -0.179 | 0.157 | -0.499 | 0.126 | 1.301 | | 0.254 |
| | **Phase (STR)[(3)]** | **-0.311** | **0.109** | **-0.53** | **-0.105** | **-2.852** | | **0.004** |
| | Phase (POST)[(3)] | -0.059 | 0.108 | -0.268 | 0.16 | -0.55 | | 0.582 |
| | Test[(2)] | 0.065 | 0.046 | -0.027 | 0.159 | 1.413 | | 0.158 |
| Precision | (Intercept) | 1.617 | 0.125 | 1.438 | 1.905 | | | - |
| | Treatment[(2)] | 0.223 | 0.174 | -0.122 | 0.547 | 1.631 | 1 | 0.202 |
| **Full model *ear low*** | | | | | | | | |
| **Part** | **Effects** | **Coefficients** | **SE** | **$CL_{lower}$** | **$CL_{upper}$** | **$\chi^2$** | **df** | ***p*** |
| Mean | (Intercept) | -2.559 | 0.55 | -3.993 | -1.624 | | | - |
| | Treatment[(2)] | 0.293 | 0.51 | -0.832 | 1.45 | | | - |
| | Phase (STR)[(3)] | 2.218 | 0.519 | 1.33 | 3.422 | | | - |
| | Phase (POST)[(3)] | 0.938 | 0.471 | -0.011 | 1.886 | | | - |
| | Test[(2)] | -0.234 | 0.153 | -0.596 | 0.068 | 2.41 | 1 | 0.120 |
| | Treatment:Phase[(4)] | | | | | **24.324** | **2** | **< 0.001** |
| | Treatment[(2)] × Phase (STR)[(3)] | -3.431 | 0.765 | -5.404 | -2.013 | | | - |
| | Treatment[(2)] × Phase (POST)[(3)] | -1.677 | 0.699 | -3.181 | -0.215 | | | - |
| Precision[(7)] | | | | | | | | - |

[(1)] not shown because of having a very limited interpretation [(2)] dummy coded ('reactive' as reference category) [(3)] dummy coded (PRE as reference category) [(4)] the indicated test refers to the overall effect of the interaction between treatment and phase [(5)] overdispersion; SE, z- and *p*-values corrected for overdispersion (*p*-value based on Wald's z-approximation; recognizable by no df indicated) [(6)] no *p*-values available due to convergence problems [(7)] no precision part because of binomial distribution of the *ear low* model
